# Clopidogrel-induced thrombotic thrombocytopenic purpura: a case report

**DOI:** 10.4314/ahs.v23i1.62

**Published:** 2023-03

**Authors:** Chidozie N Ndulue, Nonyelum N Jisieike-Onuigbo, Nwabueze J Okwesa, Arinze Anyanor, Bartholomew C Ozuemba, Nwamaka Osakwe, Fidelis Oguejiofor, Hyacinth Eze, Anya Ofia Kalu, Charles U Odenigbo

**Affiliations:** Department of Medicine, Nnamdi Azikiwe University Teaching Hospital Nnewi, Anambra State, Nigeria

**Keywords:** Clopidogrel, Thrombotic Thrombocytopenic Purpura, CKD, AKI, uremic encephalopathy

## Abstract

Thrombotic thrombocytopenic purpura (TTP) is a rare variant of thrombotic microangiopathy. We report a case of TTP in a Nigerian chronic kidney disease (CKD) patient who was previously on clopidogrel. The features of TTP resolved soon after clopidogrel was withdrawn. Clopidogrel is a cardio-protective anti-platelet drug used in CKD patients at risk of dyspepsia. However, its potential to cause TTP should be recognized and considered in acute kidney injury (AKI) patients previously on clopidogrel.

## Introduction

The hallmark of Thrombotic thrombocytopenic purpura (TTP) is the systemic deposition of platelet-rich thrombi particularly within the kidneys and brain, leading to thrombocytopaenia, anaemia, acute kidney injury (AKI) and neurologic impairment^1^. It may be hereditary, due to mutations of the ADAMTS13 gene (Upshaw-Schulman syndrome); idiopathic, immune TTP; or secondary to another disease or cause (e.g., autoimmune diseases, malignancies, infections, human immunodeficiency virus (HIV), hematopoietic stem cell transplantation and drugs). Drugs more commonly associated with TTP include ticlopidine, cyclosporine, gemcitabine and quinine. We report this case to highlight TTP as a rare complication of clopidogrel.

## Case Report

A 48-year-old woman presented with breathlessness, and worsening bilateral leg swelling of 2 weeks duration. These were associated with easy fatigability, persistent hiccups, daytime drowsiness, irrational speech, vomiting of recently ingested food, and anorexia. Leg swelling was worse in the afternoon but better in the morning. It was associated with early morning facial puffiness, and oliguria. She had been previously diagnosed with hypertensive nephropathy (CKD V A^2^) 2 years earlier and had been dialysis naive (baseline eGFR – 14ml/min). She had been transfused with 3 units of whole blood in the preceding week at another hospital before presentation. Her regular medications were: Clopidogrel, Ramipril, Hydrochlorothiazide, Amlodipine, Furosemide, Allopurinol and Calcium carbonate.

On examination, she was drowsy, pale, anicteric, acyanotic with ecchymoses at sites of venipuncture on the forearm and bilateral pitting leg edema up to the knees. Temperature-36.9°C; Pulse rate-80 bpm, Blood pressure-180/100mmHg, Respiratory rate-20c/m. Apex beat was displaced to the 6^th^ left intercostal space anterior axillary line. Her heart sounds were S4, S1 & S2, with no murmurs. Her lungs were clear. Neurological examination did not reveal any lateralizing signs. Her admitting investigations are shown in Table 1.

**Table 1:** Available investigation results at admission

**Na**	**K**	**Cl**	**HCO3**	**Urea**	**Creatinine**
104 mmol/L	6.3 mmol/L	70 mmol/L	14 mmol/L	22.7 mmol/L	1028 mcmol/L
133mmol/L	4mmol/L	95mmol/L	26mmol/L	15.4mmol/L	356µmol/L
**Hct**	**RBS**		**RVS**	**HbsAg**	**Anti-HCV**
27%	134 mg/dL		Negative	Non-reactive	Non-reactive

*serum electrolytes, urea and creatinine;* **Hct** - *Hematocrit;* **RBS** – *random blood sugar;* **RVS** – *retroviral screening;* **HbsAg** – *Hepatitis B surface antigen;* **Anti-HCV** – *Hepatitis C virus antibodies.*

A diagnosis of hypertensive nephropathy complicated by uremic encephalopathy and uremic gastritis was made and she had 2 sessions of hemodialysis over a 3-day period. These sessions were uneventful and no intradialytic complications occurred. She was also continued on her usual medications, in addition to intravenous omeprazole. On the 4th day of admission, she became febrile (Temperature-38.7°C) whereas her body temperature in the previous 3 days ranged from 36.4 – 36.7°C. Despite the hemodialysis sessions, her mental status kept deteriorating even though her SEUCr results showed improving azotemia (Na - 133mmol/L, K – 4mmol/L, Cl – 95mmol/L, HCO3 – 26mmol/L, Ur – 15.4mmol/L, Cr - 356µmol/L). RBS that day was 77 mg/dL. There were no diarrhea, tremors, seizures or differential limb weakness. On examination, she was unconscious with a Glasgow Coma Score of 8/15 (EOR-4, BVR-2, BMR-2), pale, anicteric, without cyanosis, well hydrated and widespread purpura and ecchymoses on all limbs and trunk even in sites where venipuncture had not been attempted. There were no lateralizing signs on neurologic exam. Pulse rate was 108 bpm, Blood pressure-158/100mmHg, Respiratory rate-24c/m. Lungs were clinically clear and vaginal exam was normal. Fluid input/output in the preceding 24 hours was 800ml/1000ml.

A diagnosis of Thrombotic Thrombocytopenic Purpura possibly due to Clopidogrel was entertained. Clopidogrel was discontinued. Peripheral blood film showed microangiopathic hemolysis with hypochromic erythrocytes, burr cells, microspherocytes and fragmented red blood cells; no malaria was seen on thin blood film. Direct and indirect Coombs test were non-reactive; coagulation profile was normal. Her full blood count was significant for thrombocytopenia (Platelet count - 100 X 10^9^/L, Leucocyte count – 4.9 X 10^9^/L, Neutrophil – 62%, Lymphocyte – 30%, Hematocrit – 27%)

Fever subsided the next day (37.2°C) after clopidogrel was stopped; she also became conscious though with some confusion. Two days after clopidogrel was stopped, she regained full consciousness without any further sessions of HD and was able to take food orally. This clinical improvement coincided with resolution of thrombocytopenia (162 X 10^9^ platelets/L) and microangiopathic hemolysis even though azotemia was worsening during this period (Serum urea – 18.8 mmol/L, creatinine – 828 mcmol/L).

She was discharged after 18 days on admission with a raised baseline serum creatinine of ≈ 800 mcmol/L. She is currently in an incremental maintenance hemodialysis program.

## Discussion

Thrombotic Thrombocytopenic Purpura (TTP) is a severe variant of thrombotic microangiopathy characterized by thrombocytopaenia, microangiopathic haemolytic anaemia (MAHA), microvascular wall thickening and thrombotic occlusive lesions in the kidneys and brain1. Secondary TTP is more prevalent in Black Africans and common etiologies include HIV, pregnancy and autoimmune disease^2^, ^3^.

Clopidogrel is an anti-platelet drug, commonly prescribed in CKD patients to reduce the risk of cardiovascular death and stroke, especially in persons prone to dyspepsia. It has rarely been reported to induce TTP in the global literature and, to the best of our knowledge, has not been reported from Nigeria.

TTP presents with a classic pentad which facilitates diagnosis: thrombocytopaenia, microangiopathic haemolytic anaemia, fever, cerebral impairment and kidney failure1. However, all these features may not be present or, as in the index case, some may present late in the acute illness. Kidney failure, neurologic impairment and anaemia preceded the onset of fever and purpura by weeks. This mimicked uremic encephalopathy but our suspicion was aroused when her mental status did not improve after dialysis even though biochemical azotemia was on a downward trend. After ruling out other causes like dialysis dysequilibrum syndrome and other intradialysis complications, the presence of new clinical signs made us consider TTP which was supported by haematological findings of thrombocytopenia, anaemia and microangiopathic haemolysis.

It has been proposed that the diagnosis of TTP must be confirmed by ADAMTS13 activity levels less than 10%, but this assay is expensive, labor intensive and unavailable in the region where the authors practice; also it requires highly specialized results in order to generate reliable results and it may take several days before the results are obtained^4^,^5^. On account of these it has been recommended that ADAMTS13 activity levels is not mandatory to make the diagnosis of TTP and initiate treatment^6^.

Generally, the more consistent features seen in TTP are severe thrombocytopenia (<30 X 109/L) and microangiopathic hemolysis (characterized by schistocytes). Other typical parameters include elevated reticulocyte count (>120 X 10^9^/L), undetectable serum haptoglobin, high serum LDH, negative Coombs' test, and normal coagulation results. Also, severe AKI requiring dialysis is uncommon in TTP.

In the index patient, microangiopathic hemolysis was present as evident by schistocytes seen on the peripheral blood film; direct Coombs' test was negative and her coagulation profile was normal. These are in keeping with the expected findings of TTP. Unfortunately, serum LDH, haptoglobulin and reticulocyte count were not measured due to resource constraints.

Contrary to the typical presentation of TTP, she had severe AKI and mild thrombocytopenia. This mild thrombocytopenia and severe AKI are consistent with other reports of Clopidogrel-induced TTP. Zakarija et al, in a systematic review of Clopidogrel-induced TTP, observed that most cases present with mild thrombocytopaenia, microangiopathic haemolytic anaemia and severe AKI7. The patient's prior history of CKD may have also contributed to the severity of the AKI.

Unlike other forms of secondary TTP, patients with clopidogrel-induced TTP have normal plasma ADAMTS13 activity levels. This suggests that clopidogrel initiates TTP via direct endothelial cell injury resulting in release of ultra large von Willebrand factor (ULVWF), rather than via stimulation of ADAMTS13 inhibitory antibodies8. This may explain the rapid resolution of symptoms in the index patient after clopidogrel was discontinued. The rapid resolution of thrombocytopenia and improvement in clinical status after discontinuation of Clopidogrel is in broad agreement with the TTP Outcome Criteria proposed by the International Working Group for Thrombotic Thrombocytopenic Purpura^4^.

The overall survival rate of TTP has been reported to be 67%9. Early recognition and treatment of TTP is crucial in order to improve outcomes. Discontinuation of clopidogrel in this case was associated with rapid resolution of the fever and improvement in mental status. Unfortunately, it propelled her into end stage kidney disease.

## Conclusion

Thrombotic Thrombocytopenic Purpura is a close differential of uremic encephalopathy and should be suspected when the patient does not regain full consciousness after hemodialysis; the classic pentad of TTP may be absent at presentation and only present later in the course of the illness. Clinicians should have a high index of suspicion for TTP when treating patients with suspected uremic encephalopathy, especially if the encephalopathy responds poorly to repeated hemodialysis. Furthermore, the possibility of clopidogrel-induced TTP should be borne in mind when evaluating AKI patients who are on clopidogrel.
